# Effect of physical exercise on mental health risks among Chinese left-behind children: chained mediating roles of regulatory emotional self-efficacy and psychological sense of school membership

**DOI:** 10.3389/fpsyg.2026.1812389

**Published:** 2026-07-08

**Authors:** Hao Sun, Hao Su

**Affiliations:** School of Physical Education, Shangqiu University, Shangqiu, China

**Keywords:** left-behind children (LBC), mental health risks, physical exercise, psychological sense of school membership, regulatory emotional self-efficacy

## Abstract

**Objective:**

Left-behind children (LBC) refer to youth aged less than 16 years whose parents have been absent for 6 months or longer. Given the absence of parental affection, these children frequently encounter challenges leading to severe mental health concerns. This study systematically examines the impact of physical exercise on mental health risks among LBC, including potential mediating mechanisms underlying regulatory emotional self-efficacy (RESE) and psychological sense of school membership (PSSM).

**Methods:**

The study selected 1,017 junior high students in Henan Province via cluster sampling and conducted a questionnaire survey.

**Results:**

Results revealed that physical exercise exhibited a significant negative correlation with mental health risks (r = −0.600, p < 0.01). Physical exercise was significantly and positively associated with RESE (β = 0.521) and PSSM (β = 0.299), and was significantly negatively associated with mental health risks (β = −0.613). RESE and PSSM were significantly and negatively associated with mental health risks. The total indirect effect was −0.075, accounting for 12.21% of the total effect.

**Conclusion:**

These results showed that physical exercise was not only directly associated with lower mental health risks but also showed indirect effects through RESE and PSSM.

## 1 Introduction

Left-behind children (LBC) refer to youth aged less than 16 years whose parents have been absent for 6 months or longer, leaving them to live with a single parent, grandparents, other relatives, or non-relatives. Since the 1980s, China’s deepening reform and opening-up policies have accelerated urbanization and industrialization, leading to rapid growth in labor demand. Simultaneously, faced with declining agricultural income, laborers in underdeveloped regions have resorted to urban migration as a primary livelihood strategy, resulting in large-scale rural-to-urban labor transfers. Constrained by factors such as household registration systems and economic conditions (Tomsa and Jenaro, 2015), many children of such laborers have been left behind in their registered hometowns to continue schooling or daily life. This phenomenon has led to the emergence of the distinct social group of LBC, an inevitable consequence of rapid urban development.

The root cause of LBC lies in the separation of parents and children during population migration. These children are typically left in the care of single parents, grandparents, or even relatives due to prolonged parental absence (Tang et al., 2018). Research demonstrates that LBC under the guardianship of single parents or other relatives are more prone to experiencing psychological adaptation issues (Lyu et al., 2024). Furthermore, factors such as gender, age, and duration of separation are closely associated with their mental health (Xu et al., 2019; Liu C. et al., 2024).

Mental health refers to positive states of cognitive, emotional, and social functioning. For children and adolescents, who are at critical stages of personality development and physical and psychological growth, the healthy development of body and mind plays a pivotal role in growth. This aspect not only concerns individual well-being but is also closely linked to the harmonious and stable development of society.

As a significant vulnerable group, the psychological challenges faced by the large population of LBC in China have become a focal concern for both society and academia. Given the absence of parental affection and potential issues in caregiver parenting, these children frequently encounter challenges in navigating emotional attachment, expression, and social interaction, which may contribute to elevated mental health risks. A substantial body of research has documented that LBC exhibit higher levels of internalizing problems (e.g., depression, anxiety, loneliness, and inferiority) and increased risks of externalizing problems (e.g., aggressive behavior, self-harm, and suicidal ideation) compared to their non-left-behind peers (Cheng and Sun, 2015; Feng et al., 2024; Shen et al., 2024; Guo and Li, 2023). However, while these studies have established the prevalence of mental health problems in this population, they have paid less attention to the specific psychosocial mechanisms—such as emotional regulation and school belonging—that may explain how protective factors like physical exercise relate to mental health outcomes in the LBC context.

At the same time, prolonged separation from parents and a lack of emotional communication have led to a diminished sense of belonging and support among LBC (Xu et al., 2024; Li L. et al., 2024; Zhou et al., 2021). These deficits in secure attachment and emotional availability further undermine their psychological stability. These negative psychological states further trigger or exacerbate social anxiety, mobile phone addiction, and internet addiction among LBC (Wu D. H. et al., 2024; Wu R. et al., 2024; Miao et al., 2024). This scenario renders LBC more prone to externalizing problem behaviors such as aggression, hostility, and impulsivity, potentially engendering more severe mental health risks. Furthermore, risk factors within school and environmental settings may exacerbate psychological distress. Weak teacher–student relationships, insufficient peer support, and perceived social discrimination can exert significant negative impacts on mental health status (Fu et al., 2024; Sun and Fu, 2024). Together, family and school disadvantages create a cumulative risk for maladjustment. This underscores the need for more targeted psychological interventions for LBC. For instance, research indicates that teacher support can significantly alleviate depressive symptoms among non-LBC but has a weaker effect on LBC (Li et al., 2021). Thus, intervention strategies for LBC must fully account for their unique context, specifically the lack of family support.

Regulatory emotional self-efficacy (RESE) refers to an individual’s perceived confidence in effectively regulating their emotions (Bandura et al. 2003). Individuals with high levels of RESE generally exhibit strong confidence in their ability to regulate emotions and effectively navigate diverse emotional challenges (Çelikkaleli et al., 2025).

For LBC, RESE is particularly crucial as they frequently encounter negative emotional challenges such as loneliness and anxiety (Li X. et al., 2024). Previous studies have demonstrated that multiple factors—including family environment, parental emotional support, social support, and teacher support—influence the development of RESE (Christner et al., 2024; Liu L. Q. et al., 2024). Consequently, enhancing emotional regulation efficacy to improve psychological adaptation and thereby elevate emotional management has become a key direction in psychological interventions targeting LBC (Zhu and Li, 2024; Peng et al., 2025).

Belonging is one of humanity’s most fundamental needs, influencing cognition, emotions, and behavior (Baumeister and Leary, 1995). Psychological sense of school membership (PSSM) refers to an individual’s overall perception of being accepted, included, respected, and supported within school environments (Goodenow and Grady, 1993). The literature reported that high levels of PSSM are negatively associated with depression, anxiety, and stress but positively linked to academic motivation and self-efficacy (Allen et al., 2024). For LBC, school tends to serve as the most significant socialization setting outside the home. High levels of PSSM can significantly alleviate negative emotions, thereby partially compensating for the lack of family support (Yin et al., 2025).

If adolescents experience chronically low levels of PSSM, then they may develop negative emotions such as anxiety and anger. They may then seek solace through the excessive use of the Internet or mobile phones to compensate for the lack of belonging, potentially leading to other risks such as prolonged sedentary behavior or excessive screen time. Therefore, enhancing PSSM among LBC may be an effective strategy for improving psychological well-being. However, PSSM is driven by multiple factors. Positive elements such as teacher–student relationships (Cai et al., 2023) and peer support (Zou et al., 2023) enhance it, while negative factors such as academic pressure and school bullying diminish it and further threaten mental adjustment (Lin et al., 2025). Taken together, these studies confirm the complexity of PSSM and its critical role in psychological development among adolescents.

As a form of intervention, scholars widely recognize the significant value of physical exercise in promoting mental health among adolescents (Li Z. J. et al., 2024; Molcho et al., 2021). For LBC, physical exercise not only benefits physical health but also improves emotional states through various psychological mechanisms (Song and Ding, 2024; Yao et al., 2023). Physical exercise significantly decreases symptoms of depression and anxiety while enhancing subjective well-being (Yang et al., 2024; Yang and Lu, 2025) through emotional release and neuroendocrine regulation during exercise. Concurrently, physical exercise promotes mental health through mediating pathways such as the enhancement of psychological resilience, improvement in peer relationships, and increased perceived social support (Xu et al., 2025; Shang et al., 2024).

In summary, the mental health of LBC is paramount. Moreover, RESE and PSSM are key factors that influence mental well-being, while physical exercise serves as a vital means of promoting psychological and physical development. The existing research indicated that emotional experiences during exercise can enhance RESE, thereby exerting positive effects on emotions (Doménech et al., 2024). RESE helps LBC alleviate loneliness and anxiety; only after their emotional state stabilizes are LBC more willing to engage in campus interactions and gain acceptance from peers and teachers, thereby leading to the formation of PSSM. Therefore, RESE and PSSM are not independent, parallel variables, but rather a sequential chain of mediation. However, the majority of existing studies examined relationships among these variables in isolation, thus overlooking potential complex mediating pathways such as those among physical exercise, RESE, PSSM, and mental health, which require empirical testing.

Compared to general psychological indicators such as self-esteem, psychological resilience, and peer attachment, this study selected emotional regulation self-efficacy and school belonging as chained mediating variables, which better align with the characteristics of the left-behind children population. Emotional regulation self-efficacy serves as a core internal resource for individuals to cope with negative emotions, while school belonging is a key contextual protective factor for adolescents’ school adjustment. The sequential relationship between these two variables can more effectively reveal the pathways through which mental health risks emerge among left-behind students.

**Figure 1:**
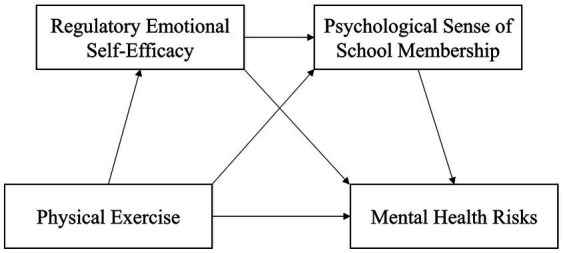
Hypothesis-based mediated model of the chained mediating effects of regulatory emotional self-efficacy (RESE) and psychological sense of school membership (PSSM) on the relationship between physical exercise and mental health risks.

Against this background, the current study systematically examines the impact of physical exercise on mental health risks among LBC, including potential mediating mechanisms underlying RESE and PSSM. Its objective is to provide concrete empirical evidence of the effectiveness of physical and mental interventions aimed toward LBC. Toward this end, the study proposes the following hypotheses. Figure 1 illustrates the hypothetical model.

*H1*: Physical exercise exerts a direct negative predictive effect on mental health risks among LBC. Specifically, greater engagement in physical exercise lowers levels of psychological distress and risk and improves mental health status.

*H2*: Physical exercise exerts a direct positive predictive effect on RESE and PSSM among LBC. Greater engagement in physical exercise increases levels of RESE and PSSM.

*H3*: RESE and PSSM exert direct negative effects on mental health risk among LBC. High levels of RESE and PSSM improve overall mental health and reduce levels of psychological distress and risk.

*H4*: RESE and PSSM exert a chained mediating effect on the relationship between physical exercise and mental health among LBC. Physical exercise sequentially enhances RESE and PSSM, ultimately decreasing mental health risks.

## 2 Materials and methods

### 2.1 Participants

The study selected five junior high schools in the eastern, southern, and northern regions of Henan Province via cluster sampling. These schools cover urban areas of county towns and township regions, including key public, general public, and private types. The locations of the selected schools are all areas with concentrated labor export in the province, where the per capita disposable income of local residents is lower than the national average, belonging to underdeveloped areas, which can well reflect the overall situation of left-behind children in counties and rural areas of Henan Province. One class was considered one sampling unit, with a certain number of administrative classes randomly drawn from each grade level. The researchers conducted preliminary background investigations to identify junior high students having one or both parents absent for more than 6 months. A total of 1,125 students were selected, and questionnaires were administered to them, yielding 1,086 responses. After excluding 69 questionnaires with systematic responses or excessive omission, 1,017 responses were considered valid (response rate: 90.4%). The respondents comprised 503 men (49.4%) and 514 women (50.6%), with 361 (35.5%) students in the 7th grade, 357 (35.1%) in the 8th grade, and 299 (29.4%) in the 9th grade, and an average age of 13.5 ± 1.1 years. The study protocol was approved by the Ethics Committee of Shangqiu University.

### 2.2 Quality control

To minimize response bias and tendencies toward habitual answering, participants were assured of the anonymity and confidentiality of their responses. All scales incorporated reverse-scored items, and uniform instructions were used for all participants. During data collection, uniformly trained teachers maintained standardized procedures through on-site supervision and guidance during the distribution and collection of questionnaires and enforced stringent time limits. Questionnaires with systematic response patterns or excessively fast completion times were excluded from the analysis.

### 2.3 Research instruments

The Physical Activity Rating Scale, revised by Liang (1994), was used to assess levels of physical activity participation among LBC. This scale evaluates physical exercise across three dimensions: intensity, duration, and frequency. Items on intensity and duration were rated using a five-point scale, while items on frequency were rated using a four-point scale. The volume of physical activity was calculated as a product of intensity, duration, and frequency, yielding a final physical exercise score ranging from 0 to 100 points.

The current study adopted the Chinese version of the Regulatory Emotional Self-Efficacy Scale, developed by Caprara et al. (2008) and revised by Wang et al. (2013). The scale comprises 17 items rated on a five-point Likert-type scale ranging from 1 (*Strongly disagree*) to 5 (*Strongly agree*). High total scores indicate high levels of RESE. In this study, Cronbach’s *α* coefficient for the total scale score was 0.92.

PSSM was measured using the Chinese version of the Psychological Sense of School Membership Scale, developed by Goodenow (1993) and revised by Zhang et al. (2021). The scale contains 16 items rated on a five-point Likert-type scale (1 = *Strongly disagree*, 5 = *Strongly agree*). High total scores indicate high levels of PSSM. In this study, Cronbach’s α coefficient for the scale reached 0.88.

The Strengths and Difficulties Questionnaire (SDQ) was used to assess psychological difficulties and prosocial behaviors among LBC. Developed by the SDQ comprises 25 items rated on a three-point scale (0 = *Does not apply* to 2 = *Applies completely*). The *difficulty score* encompasses four dimensions: emotional symptoms, conduct problems, hyperactivity/inattention, and peer relationship problems. High total scores indicate high levels of mental health risks. Additionally, higher scores on the prosocial behavior dimension represent high levels of friendly social behavior. The result of prosocial behavior subscale was not presented in the current study. Only the total difficulty score was utilized to reflect mental health risks of LBC. In this study, Cronbach’s α coefficient for the scale was 0.76.

### 2.4 Statistical analysis

Since PARS 3 uses a 0–100 scale, while RESE and PSSM use a 5-point Likert scale, all path coefficients and indirect effects in this study are reported as fully standardized coefficients (STDYX) to eliminate the impact of differing scales on the comparability of effect sizes.

Data were analyzed via SPSS 26.0. First, normality tests were conducted for the descriptive statistics of the variables, and independent sample *t*-tests were used to identify gender differences across the variables. Correlation and linear regression analyses were then performed for all variables. Finally, chained mediating effects were examined using PROCESS macro (Model 6) developed by Hayes (2013), employing the bootstrap method (5,000 repeated samples).

## 3 Results

### 3.1 Common method Bias

To assess potential bias stemming from common data sources, the study conducted unrotated exploratory factor analysis on all measurement items using Harman’s single-factor test. The results pointed to 13 factors with eigenvalues exceeding 1. The first factor explained 21.17% of variance, which falls below the 40% critical threshold (Podsakoff et al., 2003).

To further address common method bias, a marker variable test was conducted using odd-even questionnaire ID as a theoretically unrelated marker variable (Williams et al., 2010). The marker variable was included as a covariate in the mediation model to control for potential method variance. The marker variable (odd-even ID) was not significant in any regression model (*p* > 0.05). After controlling for the marker variable, all direct paths, mediation paths, and chained mediation effects remained statistically significant and consistent in effect size, indicating that common method bias did not threaten the validity of the results. Thus, no significant common method bias was identified in this study.

### 3.2 Gender differences in variables among left-behind children

Analysis of gender differences across variables (Table 1) revealed significant differences in levels of physical exercise, with male students engaging in significantly higher levels of physical exercise (*t* = 2.334, *p* = 0.020). However, gender differences did not reach statistical significance for other variables. Specifically, no significant gender difference was found for RESE (*t* = 0.647, *p* = 0.518), PSSM (*t* = 0.686, *p* = 0.493). Furthermore, male participants received lower scores on Mental Health Risks (*t* = −1.859, *p* = 0.063), but this difference did not reach statistical significance (*p* > 0.05). In addition, this study included 769 LBC with one absent parent and 248 LBC with both parents absent. An independent samples *t*-test was conducted on the mean scores of mental health risks, with left-behind status as the grouping variable. The results showed that the group with both parents absent exhibited a trend toward higher mental health risks scores, with the between-group difference reaching marginal significance (*t* = −1.758, *p* = 0.079). Although the result did not reach the statistical significance threshold of 0.05, the direction of the trend is consistent with existing literature, suggesting that the two groups of left-behind children exhibit a certain degree of heterogeneity.

**Table 1:** Gender differences in variables (*n* = 1,017).

Dimensions	Gender	*N*	M (SD)	*t*	*p*
Physical exercise	Male	503	25.02 ± 25.920	2.334	0.020^*^
	Female	514	21.42 ± 23.142		
Regulatory emotional self-efficacy			3.482 ± 0.454	0.647	0.518
			3.463 ± 0.473		
Psychological sense of school membership			3.627 ± 0.483	0.686	0.493
			3.606 ± 0.488		
Mental health risks			10.887 ± 4.667	−1.859	0.063
			11.433 ± 4.684		

*p < 0.05.

### 3.3 Correlation analyses

Spearman’s correlation analysis (Table 2) revealed that physical exercise among LBC yielded significant positive correlations with RESE (*r* = 0.454, *p* < 0.01) and PSSM (*r* = 0.268, *p* < 0.01). Additionally, physical exercise exhibited a significant negative correlation with the core dependent variable mental health risks (*r* = −0.600, *p* < 0.01). A positive correlation was found between RESE and PSSM (*r* = 0.217, *p* < 0.01), and PSSM was significantly negatively correlated with mental health risks (*r* = −0.282, *p* < 0.01). The results of this correlation analysis indicate that the interrelationships among the variables meet the conditions for testing the mediating effect.

**Table 2:** Correlation coefficients of variables.

Variables	1	2	3	4
Physical exercise	1			
Regulatory emotional self-efficacy	0.454^**^	1		
Psychological sense of school membership	0.268^**^	0.217^**^	1	
Mental health risks	−0.600^**^	−0.364^**^	−0.282^**^	1

**p < 0.01.

### 3.4 Regression analyses

Linear regression models were established, with volume of physical exercise set as the independent variable, while RESE, PSSM and mental health risks were set as the dependent variables to further explore relationships among them (see Table 3).

**Table 3:** Simple linear regression results for the predictive effects of physical exercise on psychological variables.

Variable	*R* ^2^	*F*(df)	*B*	*β*	95% CI for *β*	*t*
Regulatory emotional self-efficacy	0.271	378.097 (1, 1,015)	0.010	0.521	[0.468, 0.574]	19.445
Psychological sense of school membership	0.089	99.391 (1, 1,015)	0.006	0.299	[0.240, 0.357]	9.970
Mental health risks	0.376	611.674 (1, 1,015)	−0.117	−0.613	[−0.662, −0.565]	−24.732

Standardized coefficients (*β*) and their 95% confidence intervals (CIs) were obtained from standardized linear regression in SPSS. All regression coefficients are statistically significant (*p* < 0.01).

Regression analysis revealed that physical exercise was significantly and positively associated with RESE (*β* = 0.521, 95% CI [0.468, 0.574]) and PSSM (*β* = 0.299, 95% CI [0.240, 0.357]), explaining 27.1 and 8.9% of variance, respectively. This finding indicates that physical exercise is positively associated with RESE and PSSM, which is consistent with H2. Meanwhile, physical exercise was significantly negatively associated with mental health risks (*β* = −0.613, 95% CI [−0.662, −0.565]), explaining 37.6% of the variance, which is consistent with H1.

### 3.5 Mediation analysis

**Figure 2:**
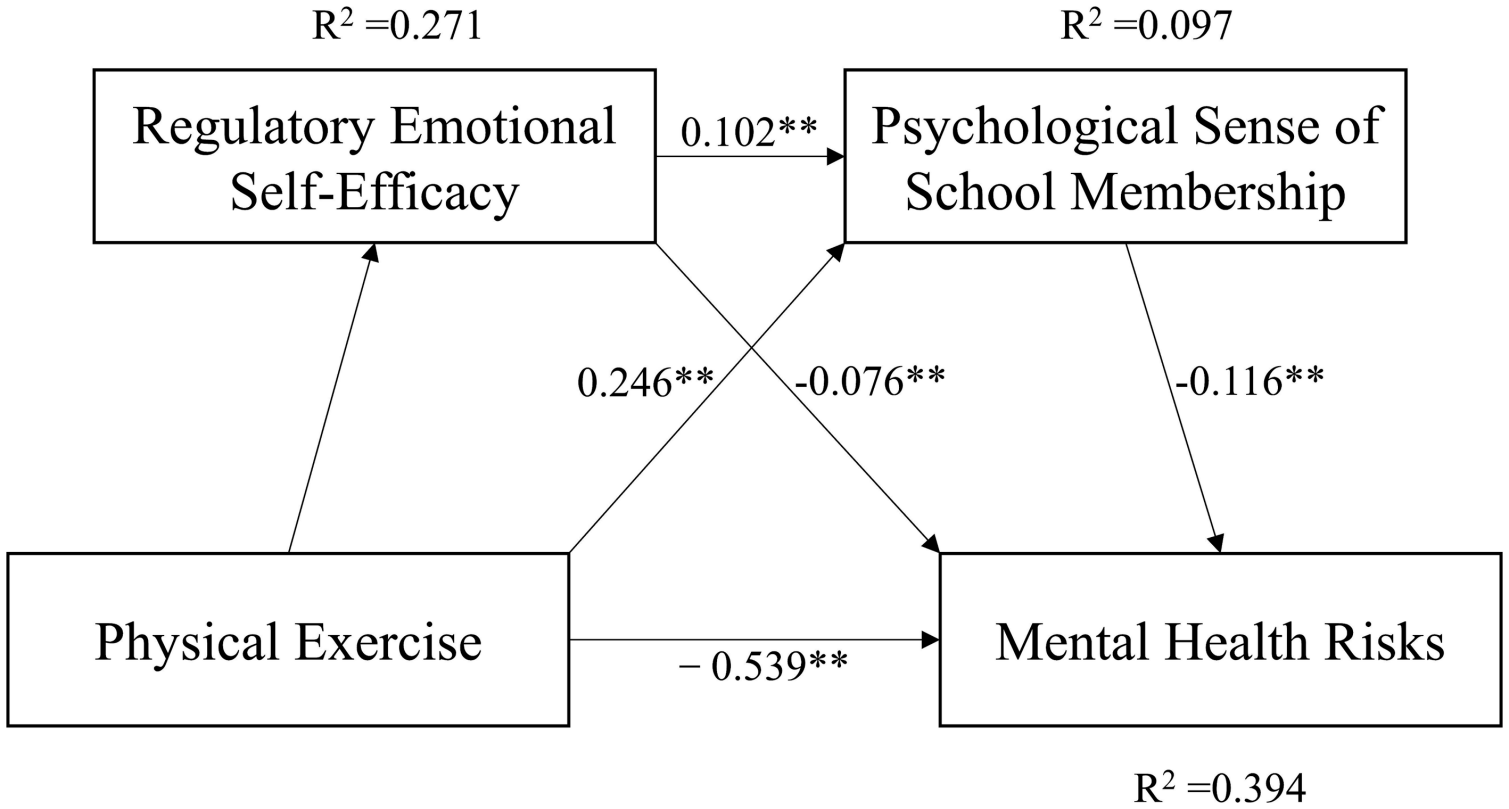
Model of the chained mediating effects of RESE and PSSM on the relationship between physical exercise and mental health risks.

After controlling for gender, physical exercise was set as the independent variable (X), Mental Health Risks as the dependent variable (Y), and RESE (M1) and PSSM (M2) as the mediating variables. A bootstrap test of mediation effects (5,000 repeated samples) was conducted, and a chained mediation model was constructed based on the results. Figure 2 illustrates the results of path analysis.

Direct effects indicate that physical exercise was significantly and negatively associated with mental health risks (Path c′: *β* = −0.539, *p* < 0.001). For the indirect path, physical exercise was significantly and positively associated with RESE (Path a1: *β* = 0.521, *p* < 0.001, 95% CI [0.468, 0.574]). RESE was significantly and positively associated with PSSM (Path d21: *β* = 0.102, *p* = 0.004, 95% CI [0.035, 0.178]). After controlling for M1, the association between physical exercise and PSSM remained significant (Path a2: *β* = 0.246, *p* < 0.001, 95% CI [0.186, 0.306]). RESE (Path b1: *β* = −0.076, *p* = 0.008, 95% CI [−0.134, −0.020]) and PSSM (Path b2: *β* = −0.116, *p* < 0.001, 95% CI [−0.161, −0.063]) were significantly and negatively associated with mental health risks.

Table 4 presents the total, direct, and three indirect effects. The total indirect effect of physical exercise on Mental Health Risks was −0.075 (95% CI [−0.109, −0.043]). The independent mediating effect of RESE (Path 1) was −0.040 (95% CI [−0.071, −0.011]). The effect size of the independent mediating role of PSSM (Path 2) was −0.029 (95% CI [−0.045, −0.015]). The effect size of the chained mediating path (Path 3) was −0.006 (95% CI [−0.012, −0.002]). These results showed that physical exercise was directly associated with lower mental health risks and also showed indirect effects through chained pathways—enhancing RESE and PSSM—and via two independent mediating pathways. The direct effect remains predominant.

**Table 4:** Indirect effects of physical exercise on mental health risks through RESE and PSSM.

Effect path	Effect value	Bootstrap *SE*	Bootstrap 95% CI
Total indirect effect	−0.075	0.017	[−0.109, −0.043]
Path 1: X → M1 → Y	−0.040	0.015	[−0.071, −0.011]
Path 2: X → M2 → Y	−0.029	0.008	[−0.045, −0.015]
Path 3: X → M1 → M2 → Y	−0.006	0.002	[−0.012, −0.002]

All effect sizes are fully standardized coefficients (STDYX).

The results of the chained mediation model suggested that the associations through which physical exercise relates to mental health are multifaceted. Physical exercise was directly associated with lower mental health risks and better mental well-being, and it also showed indirect associations through three significant indirect pathways. Taken together, these findings are consistent with the independent mediating roles of RESE and PSSM, which is consistent with H3, while the significance of the chained mediation pathways is consistent with H4. The findings indicate that for LBC, physical exercise is associated with RESE, which in turn relates to greater integration into and identification with school environments. This result revealed a statistically robust but practically small chained pathway associated with mental health development.

It should be noted that although all of the above indirect effects reached statistical significance, their effect sizes were generally small. Although all paths are statistically significant, the effect sizes are relatively small, especially the chained mediation effect. Statistical significance should be distinguished from practical significance (Funder and Ozer, 2019; Perugini et al., 2018). The total indirect effect accounted for 12.21% of the total effect, and the effect size of the chained mediation path (Path 3) was −0.006. This phenomenon stems primarily from two factors. First, the sample size in this study was large (*N* = 1,017), resulting in high statistical power, which makes it easy for even weak effects to reach significance. Second, chain mediation involves multiple steps of transmission, and each step leads to an attenuation of the effect, so the final path coefficient is typically small.

## 4 Discussion

The results indicate significant gender differences in physical exercise among LBC, with boys exhibiting significantly higher levels of physical activity (*p* < 0.05). This finding aligns with existing research reporting that men demonstrate greater willingness and engagement in sports participation (He et al., 2022; Guthold et al., 2020). This finding may stem from traditional Chinese cultural stereotypes that portray women as *reserved*, while men are expected to engage more in physical exercise, leading to higher levels of frequency and intensity in exercise. However, causal interpretation of this mechanism requires further empirical validation.

Notably, this study is based on cross-sectional data and cannot infer causality. Nevertheless, the observed associations may offer preliminary reference for school and community physical activity programs. For example, activities that align with the interests of girls, such as low-contact, high-collaboration activities (e.g., aerobic exercise, jump rope, and yoga), could be considered to lower participation barriers. In addition, organizing physical education classes or extracurricular activities led by female teachers may help create a single-gender sports environment. Peer support and role model mechanisms, such as cultivating female physical education leaders and highlighting local sports role models, may also be explored in future intervention studies.

It should be noted that the indirect effects in this study are all standardized coefficients, reflecting relative effect sizes rather than raw unit changes. The effects described above are small to moderate in magnitude; therefore, when interpreting them, the focus should be on the consistency of direction and the robustness of the statistical model, rather than placing undue emphasis on their absolute magnitude in practical terms.

Concurrently, this study did not find significant gender differences in RESE, which differs from some prior studies. Given the narrow age range of junior high school students and the unique context of family separation among left-behind children, the absence of gender differences should be interpreted with caution. This result may be related to limited developmental variability, cultural context, or the strong influence of family separation experiences, rather than gender itself. Furthermore, no significant gender differences emerged in the association between physical exercise and PSSM. This result implies that while individual behavioral patterns may exhibit gender variations, multiple factors—including cultural contexts and social support systems—moderate the psychological mechanisms underlying PSSM (Liu et al., 2025).

For mental health, male LBC exhibited lower levels of mental health risks than female LBC, although the difference was not statistically significant (*p* > 0.05). This finding aligns with previous studies that reported no significant gender differences in psychological symptoms among LBC (Fan et al., 2010). Female LBC yielded higher scores on internalizing problems such as depression, which may be related to their thought patterns and tendency to internalize emotions. This finding indicates that mental health risks among LBC may be a variable influenced by factors such as family structure and social support, with other stronger influences potentially suppressing gender effects.

Correlation analysis revealed a significant positive association between physical exercise and RESE (*r* = 0.454, *p* < 0.01). This finding is consistent with the view that physical exercise is associated with RESE via psychological capital and self-control (Zhang et al., 2023; Biddle et al., 2019). In this context, regular and appropriate physical exercise may contribute to stable physiological rhythms, and a consistent sense of accomplishment may relate to greater confidence in managing negative emotions. This result is also consistent with research showing that physical exercise is associated with fewer externalizing problem behaviors, better self-esteem and peer relationships, and higher subjective well-being (Iwon et al., 2021). Particularly for LBC, who may experience significant negative emotions such as anxiety and low self-esteem, positive experiences in physical exercise may be associated with lower trait anxiety and lower anxiety levels (Luo et al., 2023; Valtolina and Colombo, 2012).

Physical exercise also exhibited a significant positive correlation with PSSM (*r* = 0.268, *p* < 0.001), which is consistent with the conclusion that PSSM is associated with a protective effect on students’ mental health (Osterman, 2000; Slaten et al., 2016). Simultaneously, PSSM exhibited significant negative correlations with mental health risks (*r* = −0.282, *p* < 0.001). These correlations are consistent with the theoretical protective value of PSSM. However, its independent mediating effect (X → M2 → Y) was relatively small (effect size: −0.029). This finding suggests that LBC frequently face multiple risks, including emotional deprivation at home and separation anxiety (Fan et al., 2010). Therefore, future studies that explore mental health mechanisms in this population must comprehensively examine PSSM within broad family and social ecological contexts.

The results of regression analysis further indicate that physical exercise was significantly negatively associated with mental health risks (*β* = −0.613, *p* < 0.001), which accounted for 37.6% of the model variance. This study found that the effect size of the chained path through which physical exercise is associated with mental health risks among left-behind children via “RESE and PSSM” was −0.006 (95% CI: [−0.012, −0.002]). In this model, the direct effect of physical exercise on mental health risks was significant (−0.539), accounting for 87.8% of the total effect (−0.613). In line with the recommendations of relevant scholars (Funder and Ozer, 2019; Perugini et al., 2018), although this study features a large sample size and sufficient statistical power—which makes it easy for even weak associations to reach statistical significance—the effect size of the chained mediation path is −0.006, indicating an effect of limited practical significance. Not only that, the indirect and chained mediation effects are statistically significant but small in magnitude. Statistical significance does not equal practical importance, particularly in large samples. Therefore, the findings of this study should be interpreted as a statistically robust but practically limited psychosocial pathway, rather than a powerful dominant mechanism. When the direct path dominates, the additional variance explained by indirect paths (especially multi-step transmission paths) is limited. Therefore, although the effect size is small, the bootstrap confidence interval does not include zero, indicating that this chain path is robust and reliable.

It should be noted that this sequential order holds particular validity in the LBC framework. For children who are not left-behind, adequate family support provides them with emotional security, allowing RESE and PSSM to develop in parallel or influence one another causally. However, LBC, having long lacked immediate emotional responsiveness from their parents, are more sensitive to interpersonal rejection and face greater difficulties and risks in initiating social interactions. If they attempt to integrate into school before developing sufficient emotional regulation skills, encountering social setbacks may further diminish their sense of belonging at school. Therefore, for LBC, RESE serves as a prerequisite for PSSM, which aligns with the psychological adaptation patterns of this group under conditions of emotional deprivation.

This finding aligns with previous studies reporting that physical exercise is associated with fewer depressive symptoms, greater psychological resilience, and better self-awareness (Dale et al., 2019; Bourke et al., 2021).

For LBC, physical exercise may hold even greater significance. Against the background of prolonged emotional deprivation from parents, physical exercise may be one of the few daily routines that LBC can independently control (Guo et al., 2025). The resulting sense of mastery may be associated with better emotional well-being and may generalize to other life domains, which may contribute to positive psychological resource compensation. Simultaneously, the sense of accomplishment from achieving goals during exercise may be associated with a positive psychological feedback loop that may partially relate to buffering structural deficiencies in family functioning. From a social interaction perspective, physical exercise is associated with interpersonal communication and peer support, which are associated with lower social anxiety. The combined associations of these mechanisms may be related to lower psychological risks (Jung et al., 2025).

Chain mediation analysis further revealed multiple mechanisms through which physical exercise influences mental health. Physical exercise directly decreases mental health risks while exerting effects via three significant indirect pathways: an independent pathway through enhancing RESE (effect size = −0.040), an independent pathway through enhancing PSSM (effect size = −0.029), and a chained pathway through sequentially enhancing RESE and PSSM (effect size = −0.006). This finding suggests that physical exercise is associated with RESE, which in turn relates to social connectedness and PSSM, and is further associated with lower psychological risks.

To control for potential confounding effects of left-behind status and test the robustness of the model’s conclusions, this study re-examined the findings by incorporating left-behind status as a control variable in the chain mediation model. The results showed that the associations of left-behind status on RESE, PSSM, and mental health risks were all insignificant (*p* > 0.05); however, the significance and direction of the direct effects, independent mediation effects, and chain mediation effects—which form the core of this study—remained unchanged. In summary, although there was a slight trend toward baseline differences among children of different left-behind types, the chained mediation pathway through which physical exercise is associated with mental health demonstrated stability across groups, and conducting a combined overall mediation analysis is both scientifically sound and reasonable.

Notably, although the independent mediating effect of PSSM is significant, its effect size remains relatively small, indicating that the contribution of this pathway to the reduction of mental health risks through physical exercise is minimal. A potential reason is that the formation of belonging is highly dependent on social–emotional support (Baumeister and Leary, 1995). Therefore, the full realization of PSSM’s protective role among LBC may be influenced by other family-related factors. Nevertheless, this finding highlights the value of the chained mechanism identified in this study: physical exercise is significantly associated with PSSM, indicating a more resilient psychological protective pathway.

In summary, this study found associations between physical exercise and mental health among LBC. By constructing a chained mediation model that integrates RESE and PSSM, it suggests that physical exercise is associated with emotional regulation abilities and school identity, which may relate to lower psychological risks among LBC through a comprehensive pathway. However, given the cross-sectional design, causal inferences cannot be drawn. Nevertheless, the observed associations may offer preliminary reference for designing appealing physical activities within school settings and creating supportive group exercise environments, with the goal of increasing exercise participation and enhancing psychosocial adaptation among LBC.

## 5 Conclusion

This study examined the associations between physical exercise and mental health risks among LBC and explored its underlying correlates. The results point to significant gender differences in levels of physical exercise, with boys engaging in more physical activity than girls. Physical exercise was positively correlated with RESE and PSSM and negatively correlated with mental health risks. Regression analysis revealed that physical exercise was significantly associated with mental health risks. The chained mediation model suggests that physical exercise shows both direct and indirect associations with mental health risks through independent and chained pathways involving RESE and PSSM.

### 5.1 Limitations

Several limitations should be noted.

First, this study employs a cross-sectional design, which only reveals statistical associations among variables and cannot determine causal directions or causal effects. Future studies adopting longitudinal, cross-lagged, or experimental designs are needed to verify causal relationships.

Second, the sample is restricted to junior high school students in Henan Province, which may limit the generalizability of findings to other regions or age groups. Variations in population mobility, family economy, and educational resources across provinces may lead to different psychological development patterns among left-behind children.

Third, the physical activity scale (PARS-3) is a traditional self-report scale that does not fully capture new forms of digital-era physical activities. Future research may adopt more contemporary or objective measurements to improve measurement accuracy.

Fourth, this study did not include contextual factors such as peer relationship quality, teacher support, or school climate, which may restrict the explanatory power of the model. Future research may integrate more ecological factors to construct a more comprehensive framework.

Fifth, all data were collected via self-report questionnaires, which may involve common method bias and self-report bias. Although the marker variable test indicated no significant bias, multi-source and multi-method assessments are still recommended for future studies.

Finally, the chained mediation effect is statistically significant but small in magnitude, which should be interpreted cautiously by distinguishing statistical significance from practical importance, especially given the large sample size.

## Data Availability

The raw data supporting the conclusions of this article will be made available by the authors, without undue reservation.
